# Supplementary figures and images for: Characterization of resistance to a potent d-peptide HIV entry inhibitor
Source: Retrovirology. 2019 Oct 22;16:28. doi: 10.1186/s12977-019-0489-7 (PMC6805555; doi:10.1186/s12977-019-0489-7)

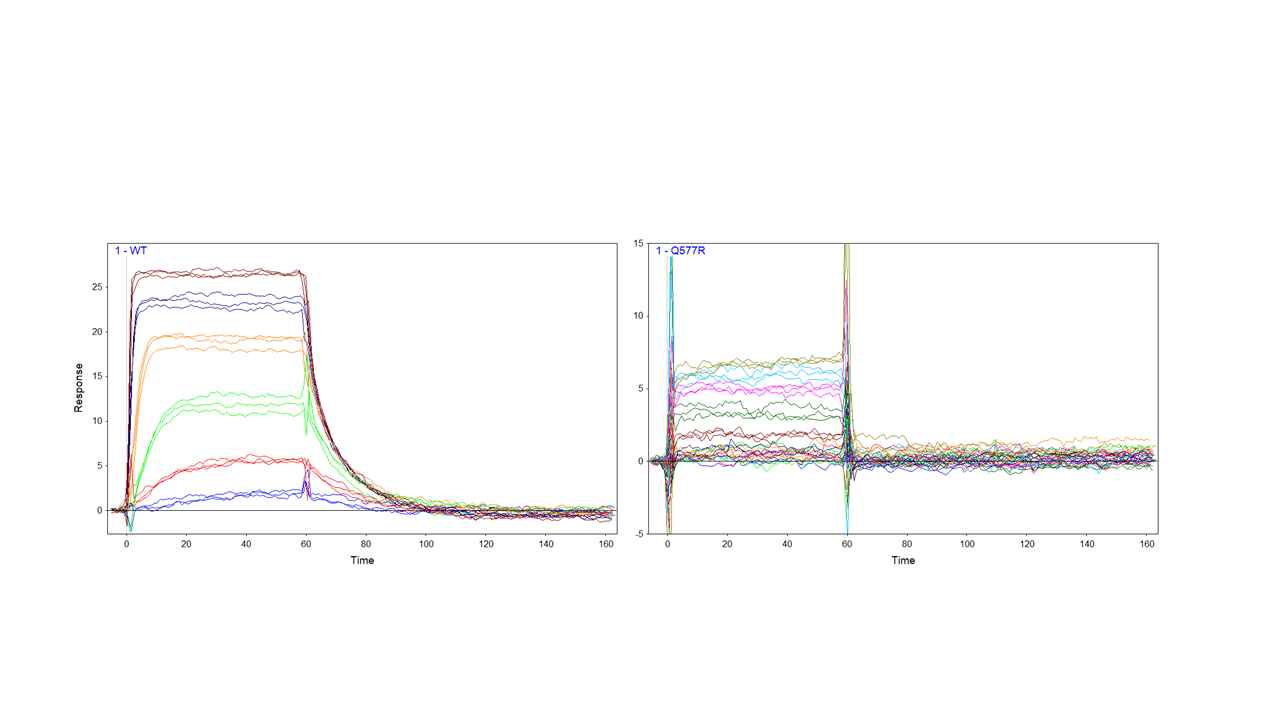

Supplement: Supplementary file 2 — Additional file 2. SPR sensorgrams for PIE12 monomer binding to IZN36 WT (left panel) or Q577R (right panel), processed in Scrubber2 (BioLogic Software) and used for the equilibrium fit shown in Fig. 2. Six threefold dilutions of PIE12 monomer (617 nM to 2.54 nM) were flowed over the WT surface, and ten threefold dilutions (50 µM to 2.54 nM) were flowed over the Q577R surface. The calculated KD’s are 0.031 µM for WT and 2.0 µM for Q577R. [file 12977_2019_489_MOESM2_ESM.tif]
