# Supplementary material for: Characterization of resistance to a potent d-peptide HIV entry inhibitor
Source: Retrovirology. 2019 Oct 22;16:28. doi: 10.1186/s12977-019-0489-7 (PMC6805555; doi:10.1186/s12977-019-0489-7)
Supplement: Supplementary file 3 — Additional file 3. Effect of Q577R on C-peptide Inhibitors. Single-cycle viral infectivity assays in which HIV-1 HXB2 Env (WT and Q577R) pseudotyped HIV-1 with a luciferase reporter was used to infect HOS-LES cells in the absence or presence of six fivefold dilutions of the indicated C-peptide (in quadruplicate). The data are the average of two experiments with the standard deviation in parentheses. [file 12977_2019_489_MOESM3_ESM.pdf]

	IC50 (nM)		
	WT	Q577R	Fold Difference
C34	0.355 (0.177)	1.58 (0.30)	4.8
T20	1.62 (0.23)	5.33 (0.74)	3.4
T1249	0.152 (0.064)	0.18 (0.05)	1.2
