# Supplementary material for: Characterization of resistance to a potent d-peptide HIV entry inhibitor
Source: Retrovirology. 2019 Oct 22;16:28. doi: 10.1186/s12977-019-0489-7 (PMC6805555; doi:10.1186/s12977-019-0489-7)
Supplement: Supplementary file 4 — Additional file 4. Prevalence of PIE12-trimer resistant candidate compensatory amino acid mutations in Group M primary isolates containing Q577R. [file 12977_2019_489_MOESM4_ESM.docx]

| WT Position | PIE12-trimer resistant Mutation | Prevalence in Q577R containing Primary Isolates | Other mutations observed at this position | Comments |
| --- | --- | --- | --- | --- |
| A48 | A48**T** | 1/751 | None | - Alanine is conserved in 750/751 (99.9%) sequences at this position |
| 161-164 amino acids: ISTS | Δ161-164 | 0/751 |  | - Loss of a glycosylation site with this deletion - 724/751 sequences have the intact glycosylation site |
| 396-400  amino acids: FNSTW | Δ396-400 | 0/751 |  | - Loss of a glycosylation site with this deletion - This region is highly variable - 16/751 lack at least one of the V4 glycosylation sites in this region |
| Q550 | Q550**H** | 0/751 |  | - The WT Q550 is conserved in all 751 sequences. Twenty-two have the cag codon; 729 have caa. |
| V583  Codon: GTG | V583  Codon: GT**A** | 28/751 | V583I, V583L and V583M (168/751) | - 583/751 sequences have V583. Of these, 28 have the gta codon. - Only hydrophobic residues are seen at this position in this viral pool - All variants at this position (Val, Ile, Leu and Met) have a T as the second position in the codon - In the RRE structure, that T(U) is in a G-U pair at the end of a stem-loop—suggests this T(U) is important |
| L663 | L663**F** | 1/751 | L663W (2/751) | - 748/751 have WT L663 |
| A823 | A823**V** | 0/751 | A823G (134/751) | - 617/751 have WT A823 |
